# Downregulation of TCF1 in HIV Infection Impairs T-cell Proliferative Capacity by Disrupting Mitochondrial Function

**DOI:** 10.3389/fmicb.2022.880873

**Published:** 2022-07-06

**Authors:** Hong-Jiao Cai, Jue Shi, Lin-Bo Yin, Jie-Fu Zheng, Ya-Jing Fu, Yong-Jun Jiang, Hong Shang, Zi-Ning Zhang

**Affiliations:** ^1^NHC Key Laboratory of AIDS Immunology, National Clinical Research Center for Laboratory Medicine, The First Affiliated Hospital of China Medical University, Shenyang, China; ^2^Department of Central Laboratory, Dalian Municipal Central Hospital, Dalian, China; ^3^Key Laboratory of AIDS Immunology, Chinese Academy of Medical Sciences, Shenyang, China; ^4^Department of Laboratory Medicine, Zhuhai Hospital of Integrated Traditional Chinese and Western Medicine, Zhuhai, China

**Keywords:** HIV infection, mitochondrial function, T-cell factor 1, proliferative capacity, metabolism

## Abstract

**Background:**

Despite the benefits of antiretroviral therapy (ART) for people with HIV, T-cell dysfunction cannot be fully restored. Metabolic dysregulation is associated with dysfunction of HIV-1-specific T-cells. Exploration of the factors regulating metabolic fitness can help reverse T-cell dysfunction and provide new insights into the underlying mechanism.

**Methods:**

In this study, HIV-infected individuals and HIV-negative control individuals (NCs) were enrolled. T-cell factor (TCF)1 expression in cells was determined by quantitative reverse-transcriptase polymerase chain reaction and flow cytometry. Relevant microarray data from the GEO database were analyzed to explore the underlying mechanism. The effects of TCF1 on T-cell function and metabolic function were assessed *in vitro*.

**Results:**

*TCF7* mRNA expression in peripheral blood mononuclear cells was downregulated in rapid progressors compared with long-term non-progressors individuals and NCs. TCF1 expression on CD4^+^ and CD8^+^ T-cells was downregulated in treatment-naïve HIV-infected individuals compared with NCs. Interleukin (IL)2 production and proliferative capacity were impaired in TCF1 knockdown T-cells. Moreover, glycolytic capacity and mitochondrial respiratory function were decreased in TCF1 knockdown T-cells, and depolarized mitochondria were increased in TCF1 knockdown T-cells.

**Conclusion:**

Downregulation of TCF1 in HIV infection impairs T-cell proliferative capacity by disrupting mitochondrial function. These findings highlight the metabolic regulation as a pivotal mechanism of TCF1 in the regulation of T-cell dysfunction.

## Introduction

During chronic viral infections including HIV, T-cell exhaustion occurs due to chronic exposure to antigens, inflammatory signals, lack of CD4^+^ T-cell helper cells, and/or cell-intrinsic defects (Day et al., 2006; Fenwick et al., 2019). T-cell exhaustion is characterized by progressive loss of cell proliferation and effector functions, metabolic dysregulation, increased inhibitory receptor expression, and distinct transcriptional signatures (Kurachi, 2019; McLane and Wherry, 2019). Despite the benefits of ART for people with HIV, these deficiencies cannot be fully restored (Rehr et al., 2008; Migueles et al., 2009).

Metabolic dysregulation during chronic HIV infection, including reductions in glucose uptake, progressive mitochondrial damage, and increased reactive oxygen species (ROS) production, likely contributes to accelerated T-cell aging, senescence, and apoptosis (Bengsch et al., 2016; Desdin-Mico et al., 2018; McLane and Wherry, 2019). Accumulating evidence supports the therapeutic potential of targeting exhausted T (Tex) cells, for example *via* inhibitory receptor blockade, thus increasing glucose uptake and mitochondrial fitness and reinvigorating Tex cells (Bengsch et al., 2016; Masao Hashimoto et al., 2018; Saeidi et al., 2018; McLane and Wherry, 2019). Therefore, identifying the mechanism that leads to metabolic dysfunction, which ultimately results in T-cell exhaustion, is essential to explore effective and biologically plausible immunotherapeutic interventions for controlling disease progression.

T-cell factor 1 (TCF1, encoded by *TCF7*) is a key transcription factor that regulates T-cell development and proliferative capacity (Weber et al., 2011; Sharma et al., 2012) by initiating the canonical WNT and NOTCH signaling pathways (Germar et al., 2011; Escobar et al., 2020). In models of chronic viral infection, studies have revealed that TCF1^+^ T-cells represent a population of stem-like or progenitor exhausted T-cells (Tpex; Snell et al., 2018; Chen et al., 2019). Only TCF1^+^ T-cells, unlike their TCF1^−^ counterparts, have the ability to self-renew and give rise to a progeny of terminally exhausted TCF1^−^ cells with effector potential (Siddiqui et al., 2019). TCF1 overexpression in CD8^+^ tumor-infiltrating lymphocytes (TILs) enhanced cytokine-producing capacity and suppressed co-inhibitory receptor expression while retaining a heightened response to checkpoint blockade, leading to enhanced tumor control (Kurtulus et al., 2019; Siddiqui et al., 2019; Shan et al., 2020). In clinical trials of individuals with melanoma, an increased frequency of TCF1^+^CD8^+^ T-cells expression was found to be positively correlated with patient survival and responded well to checkpoint-blockade therapy (Miller et al., 2019; Sade-Feldman et al., 2019). HIV-specific CD8^+^ T-cells expressing TCF1 were highest in “elite controllers” who can naturally control viral load below the detection limit without ART, followed by ART-suppressed and then HIV^+^ viremic individuals (Takuya Sekine AP-P, 2020; Rutishauser et al., 2021). Furthermore, TCF1 contributes to the regulation of the expansion capacity of HIV-specific CD8^+^ T-cells (Rutishauser et al., 2021). Recent research found that Tpex with high TCF1 expression could sustain mitochondrial fitness over time (Gabriel et al., 2021), but the specific role of TCF1 in regulating the metabolic fitness and how TCF1 deletion contributes to metabolic dysfunction or impairs cellular function in chronic HIV infection, is still not entirely clear.

Given that TCF1 plays an important role in regulating T-cell development and proliferative capacity, we hypothesized that TCF1 may act as a protective factor in HIV infection. In this study, we tested our hypothesis by investigating the relationship between TCF1 expression level, viral load, and CD4^+^ counts. We also studied the effects of TCF1 in T-cell function and metabolic function. Our results demonstrate that lower TCF1 expression with HIV infection could impair T-cell function *via* mitochondrial damage.

## Materials and Methods

### Study Population and Recruitment

A total of 56 HIV-infected individuals and 55 HIV-negative control individuals (NCs) were included in our study. Of these, in our analysis of *TCF7* mRNA expression in peripheral blood mononuclear cells (PBMCs), eight “rapid progressors” (RPs; CD4^+^ T-cell<350 cells/ul within 1–2 years of HIV infection; 8 males; average age is 48 years), seven “long term non-progressors” (LTNPs; individuals who maintained normal CD4^+^ T counts and controlled viremia efficiently for prolonged periods after HIV infection; 5 males and 2 females; average age is 51 years), and seven aged- and sex- matched NCs were enrolled. In the analysis of *TCF7* mRNA expression in CD4^+^ and CD8^+^ T-cells, seven HIV-infected patients (7 males; average is 45 years) and five aged- and sex- matched NCs were included. To analyze TCF1 expression in T-cells using flow cytometry, 16 HIV-infected individuals receiving ARTs, seven treatment-naïve HIV-infected individuals (HIVs), and 11 NCs were enrolled; to detect interleukin (IL)2 production, we included 18 individuals; and to evaluate the proliferative capacity of T-cells, we included 12 individuals. Finally, we included 13 individuals for a functional and metabolic analysis of TCF1 knockdown by small interfering (siRNA).

All individuals included in our study signed informed consent forms before participating in this research project. The study was approved by the Research and Ethics Committee of the First Affiliated Hospital of China Medical University, Shenyang, China.

### Preparation of Cells

Whole blood samples were collected in EDTA vacutainers (BD, New Jersey, United States) to obtain PBMCs by density gradient centrifugation. Human T-cells were isolated from PBMCs using human T-cells negative isolation kit (StemCell Technologies, Vancouver, Canada). Cell purity was >96% confirmed by flow cytometry (Supplementary Figure 1).

### RNA Reverse Transcription and Quantitative Real-Time Polymerase Chain Reaction

Quantities of *TCF7* mRNA in PBMCs, CD4^+^ T-cells, and CD8^+^ T-cells were determined using qRT-PCR. Total RNA was first extracted using the RNeasy RNA isolation kit (Qiagen, Stanford, VA, United States) and was reverse transcribed into complementary DNA (cDNA) using the PrimeScript^™^ RT reagent kit (TaKaRa Biotechnology) according to the manufacturer’s instructions. Expression levels of *TCF7* mRNA were evaluated using TB Green Premix Ex Taq™ II (TaKaRa Biotechnology) on Roche LightCycler^®^480 Real-Time PCR system with the following primers synthesized by BGI (Beijing, China):

*TCF7*-F:5′-CCTTGATGCTAGGTTCTGGTGTACC-3′

*TCF7*-R:5′-CACTCTGCAATGACCTTGGCTCTCA-3′

The housekeeping gene GAPDH was included as an internal standard. *TCF7* mRNA expression was measured in duplicate and calculated *via* the Livak method.

### TCF1 Knockdown in T-cells Using siRNA

To investigate the effects of TCF1 on T-cell functions, 200 pmol *TCF7-*siRNA or siRNA Negative Control (Thermo Fisher Scientific, Waltham, MA, United States) was transfected into separate CD3^+^ T-cells, respectively, using the Human T-cell Nucleofector^®^ Kit (Lonza) according to the manufacturer’s protocol. Cells were then incubated for 24 h post Nucleofection. For transfection efficiency detection, quantities of *TCF7* mRNA were performed after transfection for 24 h, as described in the methods section of RNA reverse transcription and qRT-PCR.

### Staining and Flow Cytometric Analysis

To investigate TCF1 expression in T-cell subsets, isolated cells were labeled with antibodies against CD3, CD4, CD8, CCR7, CD45RA, PD-1, and TIGIT (Biolegend) for 20 min at 4°C. Subsequently, Fixation/Permeabilization working solution (eBiosciences) was added, and the cells were followed by incubation with anti-TCF1 (BD Biosciences) for 30 min at room temperature. To investigate cytokine production in TCF1^+^ and TCF1^−^ T-cell, isolated T-cells or transfected cells were stimulated with Dynabeads™ Human T Activator CD3/CD28 (beads to cell ratio, 1:2; Thermo Fisher) for 24 h. GolgiStop (1 μl/mL, BD Biosciences) was added to the culture for the final 6 h. After that, cells were stained with LIVE/DEAD™ Fixable Aqua Dead Cell Stain kit (Invitrogen) for 30 min at 4°C. The cells were then labeled with antibodies against CD3, CD4, CD8, anti-TCF1, anti-IL-2, and anti-IFN-γ, as described above. To investigate the effects of TCF1 on T-cell proliferation, isolated T-cells or transfected cells were marked with Cell Trace™ Violet (Thermo Fisher) for 30 min at 37°C. After incubation at 37°C for 3 days, cells were incubated with antibodies directed against LIVE/DEAD™ Fixable Aqua Dead Cell Stain kit, CD3, CD4 CD8, and anti-TCF1, as described above. To detect mitochondrial mass (MM) and membrane potential (MMP), the transfected cells were resuspended in prewarmed (37°C) staining solution containing MitoTracker^®^ Green FM (50 nm; Thermo Fisher Scientific) and MitoTracker^®^ Orange CMTMRos (25 nm; Thermo Fisher Scientific) for 30 min, washed the cells with PBS and then stained with LIVE/DEAD™ Fixable Aqua Dead Cell Stain kit.

Cells were detected using the LSR II flow cytometer (BD Biosciences, San Jose, CA, United States) and data were analyzed using FlowJo software (Ashland, OR, United States).

### Analysis of Microarray Data

To explore the underlying mechanism of impaired cell function with TCF1, we downloaded microarray data from Gene Expression Omnibus (GEO)^1^ with accession number GSE44228. According to the mean expression of TCF1 in the data, we stratified the samples into two groups: TCF1^high^ and TCF1^low^. Using the online GEO2R analysis tool,^2^ we identified differentially expressed genes (DEGs) between TCF1^high^ and TCF1^low^ with *p*-value <0.05 and fold change (FC) >1.2. Functional enrichment of DEGs was conducted using Gene Ontology (GO) and Kyoto Encyclopedia of Genes and Genomes (KEGG) pathway enrichment analyses on the DAVID website,^3^ and the results were visualized using the online tool ImageGP.^4^

### Seahorse Extracellular Flux Analysis

On the day prior to this assay, we hydrated an Agilent Seahorse XFp Sensor Cartridge with XF Calibrant in a non-CO_2_ 37°C incubator overnight. Transfected cells were stimulated with ImmunoCult Human CD3/CD28 T-cell Activator (StemCell Technologies, Vancouver, Canada) for 24 h. On the day of the assay, we firstly resuspended cells in a warmed assay medium to the desired concentration (5 × 10^5^ cells in 50 μl/well) before seeding them on Cell-Tak-coated Seahorse Cell Culture Miniplate (wells A and H were background correction wells). Then, we centrifuged the cells at 350 × g (zero braking) for 5 min and added 130 μl assay medium to each well for a final volume of 180 μl. Finally, the Miniplate was transferred to a non-CO_2_ 37°C incubator for 25–30 min to ensure that the cells were entirely attached. When the above was prepared, basal and maximal respiration (OCR) and extracellular acidification (ECAR) were analyzed using an XFp Cell Mito Stress Test Kit and XFp Glycolysis Stress Test Kit on an Agilent Seahorse XF HS Mini instrument according to the corresponding procedure.

### Statistics

All statistical analyses were performed using GraphPad Prism v7.0 (GraphPad, San Diego, CA, United States). Normality tests were performed before analyzing the data. Mann–Whitney U test or Paired *t*-test were used to evaluate differences between two groups. One-way ANOVA analysis was used to compare *TCF7* mRNA and TCF1 expression levels between 3 groups. Pearson’s correlation was used to assess correlations between viral load, CD4^+^ T-cell counts, and TCF1 expression levels. Data were recorded as mean and standard deviation (SD); *p* values <0.05 were considered statistically significant.

## Results

### *TCF7* mRNA Expression Is Lower in HIV-Infected Individuals and Is Associated With Disease Progression

**Figure 1:**
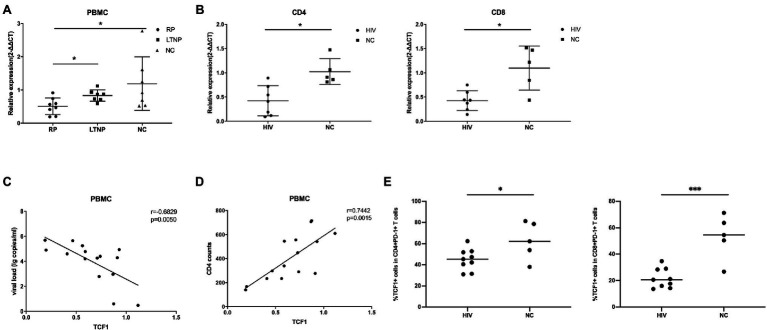
*TCF7* mRNA expression is reduced in HIV-infected individuals and is associated with disease progression. **(A)** *TCF7* mRNA expression in peripheral blood mononuclear cells (PBMCs; from eight “rapid progressors” [RPs], seven long-term non-progressors [LTNPs], and seven HIV-negative control individuals [NCs]) was quantified and normalized to that of glyceraldehyde 3-phosphate dehydrogenase (GAPDH) transcripts, and expressed using the relative quantification method (2^-∆∆Ct^). *TCF7* mRNA expression levels were compared between three groups by one-way ANOVA analysis. **(B)** *TCF7* mRNA expression in CD4 and CD8^+^ T cells from HIV-infected individuals (*n* = 7) and NCs (*n* = 5) was quantified and normalized to that of GAPDH transcripts and expressed using the relative quantification method (2^-∆∆Ct^). The Mann–Whitney U test was used for intergroup comparisons. **(C)** Pearson’s correlation analysis of *TCF7* mRNA expression and viral load in PBMCs from RPs and LTNPs (*n* = 15). **(D)** Pearson’s correlation analysis of *TCF7* mRNA expression and CD4^+^ T cell counts in PBMCs from RPs and LTNPs (*n* = 15). **(E)** The percentage of TCF1^+^ cells in CD4^+^PD1^+^ T cells and CD8^+^PD1^+^ T cells form HIV-infected individuals (*n* = 9) compared with HIV negative control individuals (*n* = 5). *p < 0.05, ***p < 0.001.

To explore the relationship between *TCF7* mRNA and HIV, we first compared the expression of *TCF7* mRNA in three groups (including eight RPs, seven LTNPs, and seven NCs). The qRT-PCR results showed that the level of *TCF7* mRNA was significantly lower in PBMCs from RP individuals than LTNPs (*p* = 0.0205) and NCs (*p* = 0.0401; Figure 1A). *TCF7* mRNA expression in PBMCs was negatively correlated with viral load (*p* = 0.0050; Figure 1C) and positively correlated with CD4^+^ counts (*p* = 0.0015; Figure 1D). In addition, *TCF7* mRNA expression was substantially lower in HIV-infected patients than in NCs, both in CD4^+^ T-cells (*p* = 0.0101) and CD8^+^ T-cells (*p* = 0.0177; Figure 1B). The expression of TCF1 on antigen-experienced CD4^+^PD-1^+^ and CD8^+^PD-1^+^ T-cells was also significantly lower in HIV-infected patients compared to NCs (*p* = 0.0328, *p* = 0.0003; Figure 1E), respectively. These results indicated that TCF1 might prevent disease progression from HIV infection to some extent.

### The TCF1 Expression in T-cells Is Significantly Lower in HIV-Infected Patients

**Figure 2:**
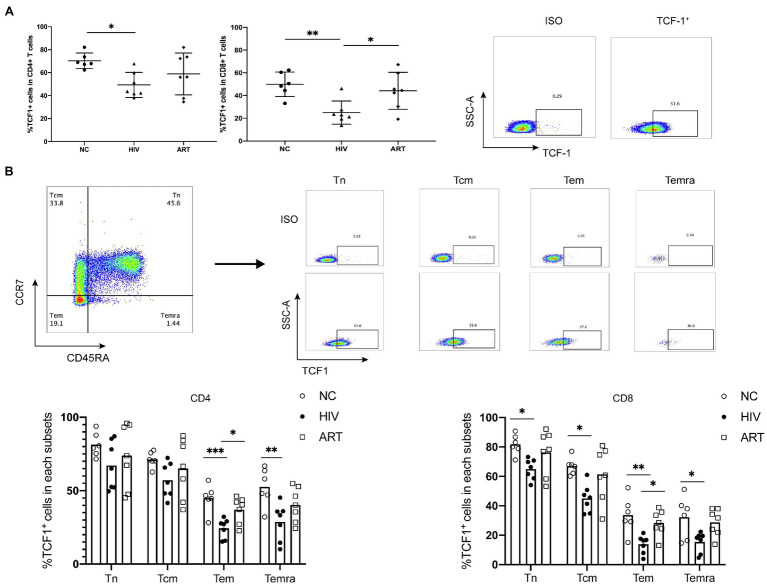
T cell factor (TCF)1 expression is reduced in different CD4^+^ and CD8^+^ T cell subsets from HIV-infected individuals. **(A)** The percentage of TCF1^+^CD4^+^ T cells and TCF1^+^CD8^+^ T cells from treatment-naïve HIV-infected individuals (HIVs, *n* = 7) compared with HIV-negative control individuals (NCs, *n* = 6) and HIV-infected individuals receiving antiretroviral therapy (ARTs, *n* = 7). The TCF1^+^ population was gated based on the ISO from the same individual. **(B)** The percentage of TCF1^+^ cells in naïve (Tn), central memory (Tcm), effector memory (Tem), and terminally differentiated effector memory (Temra) T cell populations from CD4^+^ and CD8^+^ T cells (including HIVs, NCs, and ARTs), distinguished by the level of CCR7 and CD45RA expression. One-way ANOVA analysis was used in **(A,B)**. *p < 0.05, **p < 0.01, ***p < 0.001.

To acquire comprehensive and detailed information on TCF1 protein expression, we conducted further analyses using flow cytometry. The percentage of TCF1^+^CD4^+^ T and TCF1^+^CD8^+^ T-cells was significantly lower in the HIVs compared to NCs (*p* = 0.0271, *p* = 0.0076), and the percentage of TCF1^+^CD8^+^ T-cells was significantly higher in ARTs than HIVs (*p* = 0.0319; Figure 2A). We subdivided CD4^+^ and CD8^+^ T-cells into naïve (Tn), central memory (Tcm), effector memory (Tem), and terminally differentiated effector memory (Temra) cells, based on the cell surface markers CD45RA and CCR7 (Figure 2B). We then compared the TCF1 expression on the four T-cell subsets. The results indicated that reduced TCF1 expression on CD4^+^ T-cells in HIVs predominantly occurred in Tem, and Temra cells (*p* = 0.0010, *p* = 0.0079), and on CD8^+^ T-cells in HIVs this occurred mainly appeared in Tn, Tcm, Tem, and Temra cells (*p* = 0.0265, *p* = 0.0153，*p* = 0.0034, and *p* = 0.0235; Figure 2B). We also observed that TCF1 was expressed to a greater extent on Tn cells and Tcm cells than Tem and Temra cells across all groups (Figure 2B). These results together indicate that TCF1 may act as a protective factor in HIV infection, and also that the expression pattern of TCF1 was consistent with the character of TCF1^+^ stem-like T-cells.

### Downregulation of TCF1 Impairs the Proliferative Capacity of T-cells

**Figure 3:**
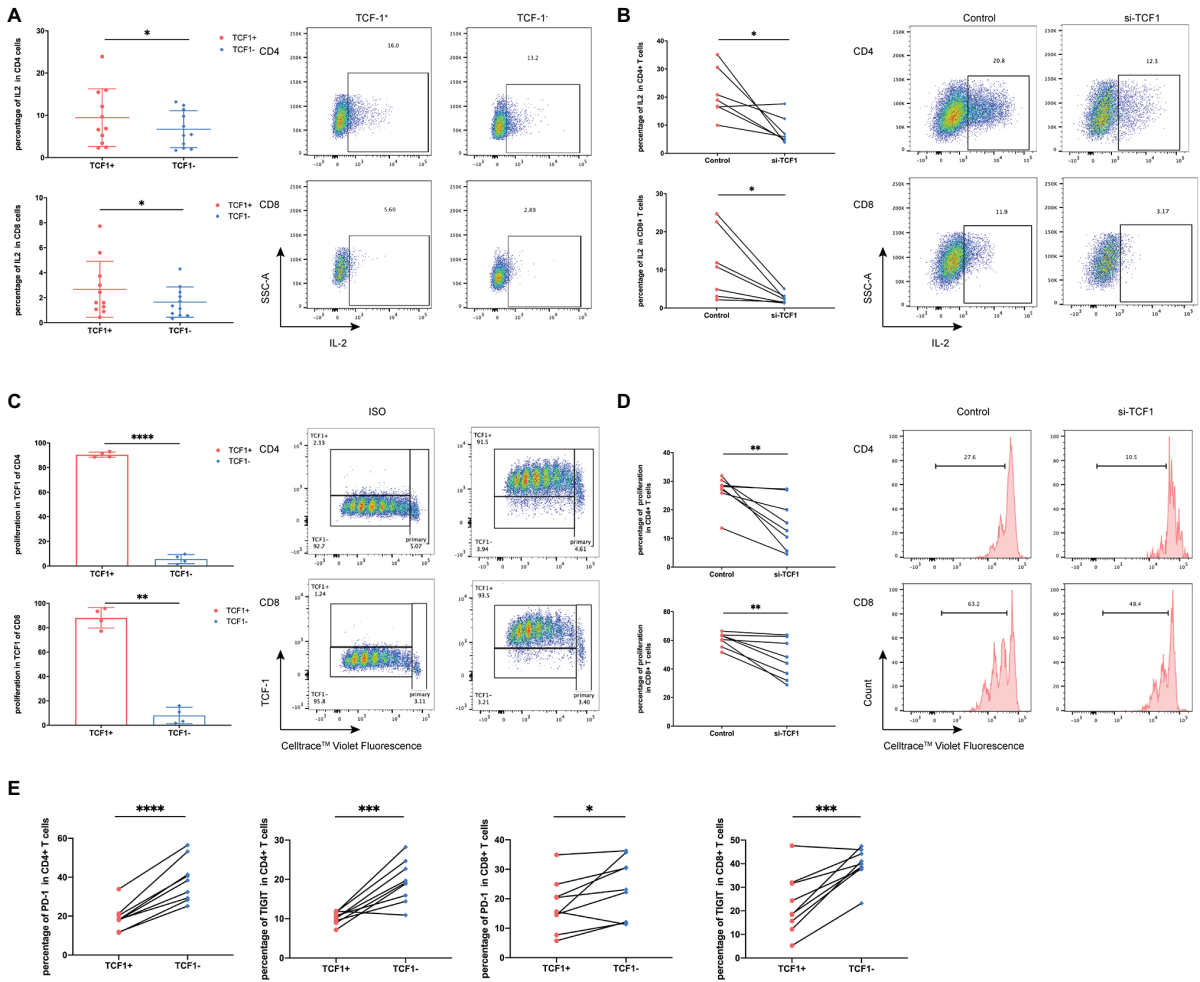
Downregulation of T cell factor (TCF)1 impairs T cell proliferation. **(A)** To investigate whether TCF1 affects T cell function, primary CD3^+^ T cells (*n* = 11) were stimulated with anti-αCD3/CD28 Dynabeads for 24 h, and GolgiStop (1 μl/ml) was added to the culture for the final 6 h. Interleukin (IL)2 expression in TCF1^+^ T cells was compared with TCF1^−^ T cells. **(B)** After 24 h transfection with siTCF1, primary T cells (*n* = 7) were stimulated using anti-αCD3/CD28 Dynabeads for 24 h and intracellular expression of IL2 was determined. **(C)** Primary CD3^+^ T cells (*n* = 4) were labeled with Cell Trace™ Violet and stimulated using anti-αCD3/CD28 Dynabeads for 72 h. The proliferation of TCF1^+^CD4^+^ and TCF1^+^CD8^+^ T cells increased significantly compared with TCF1^−^CD4^+^ and TCF1^−^CD8^+^ T cells. **(D)** After knockdown with siTCF1 for 24 h, primary CD3^+^ T cells (*n* = 8) were labeled with Cell Trace™ Violet and stimulated using anti-αCD3/CD28 Dynabeads for 72 h. The proliferation of CD4^+^ and CD8^+^ T cells decreased significantly in the absence of TCF1 expression. **(E)** PD-1 and TIGIT expression in TCF1^+^ T cells was compared with TCF1^−^ T cells (*n* = 9). Paired *t*-tests were used for analysis. *p < 0.05, **p < 0.01, ***p < 0.001.

Because TCF1 is downregulated in HIV-infected individuals, we hypothesized that TCF1 may affect T-cell function. To test this, we stimulated T-cells for 24 h with Dynabeads™ Human T Activator CD3/CD28 (Thermo Fisher) and detected the production of IL2 and IFN-γ in TCF1^+^ and TCF1^−^ cell subsets. The results showed that less IL2 was produced by TCF1^−^ compared with TCF1^+^ cells, both in CD4^+^ T-cells (*p* = 0.0186) and CD8^+^ T-cells (*p* = 0.0222; Figure 3A). As IL2 can stimulate T-cell entry into the cell cycle and thus induce cell proliferation, we analyzed proliferation capacity in TCF1^+^ and TCF1^−^ cells. The results showed that the new progeny cells mainly originated from TCF1^+^ rather than TCF1^−^ cells, both in CD4^+^ T-cells (*p* < 0.0001) and CD8^+^ T-cells (*p* = 0.0018; Figure 3C). And the percentage of new progeny cells in TCF1^−^ after 24 h was less than 10% (Figure 3C). To confirm this result, we employed *TCF7-*siRNA to knockdown TCF1 in T-cells. *TCF7* mRNA expression was significantly downregulated in T-cells transfected with siRNA (Supplementary Figure 2). The production of IL2 by CD4^+^ (*p* = 0.0205) and CD8^+^ (*p* = 0.0261) T-cells was substantially reduced after TCF1 was knocked down (Figure 3B), and the proliferative capacity of CD4^+^ (*p* = 0.0076) and CD8^+^ (*p* = 0.0084). T-cells was also impaired in the TCF1 knockdown cells (Figure 3D), confirming the results shown in Figures 3A,C. Knockdown of *TCF7* has the tendency to decrease IFN-γ production of the CD8^+^ T-cells, but it did not reach statistical significance (*p* = 0.1180; Supplementary Figure 3). These results indicate that TCF1 plays a pivotal role in sustaining the proliferative capacity of T-cells. We also detected immune checkpoints and found that the percentage of PD-1 and TIGIT was significantly lower in the TCF1^+^ cells compared with TCF1^−^ cells, both in CD4^+^ T-cells (*p* < 0.0001, *p* = 0.0009) and CD8^+^ T-cells (*p* = 0.0319, *p* = 0.0010), indicating the relationship between TCF1 expression and T-cell exhaustion (Figure 3E).

### The Glycolytic Function Was Decreased in TCF1 Knockdown Cells

**Figure 4:**
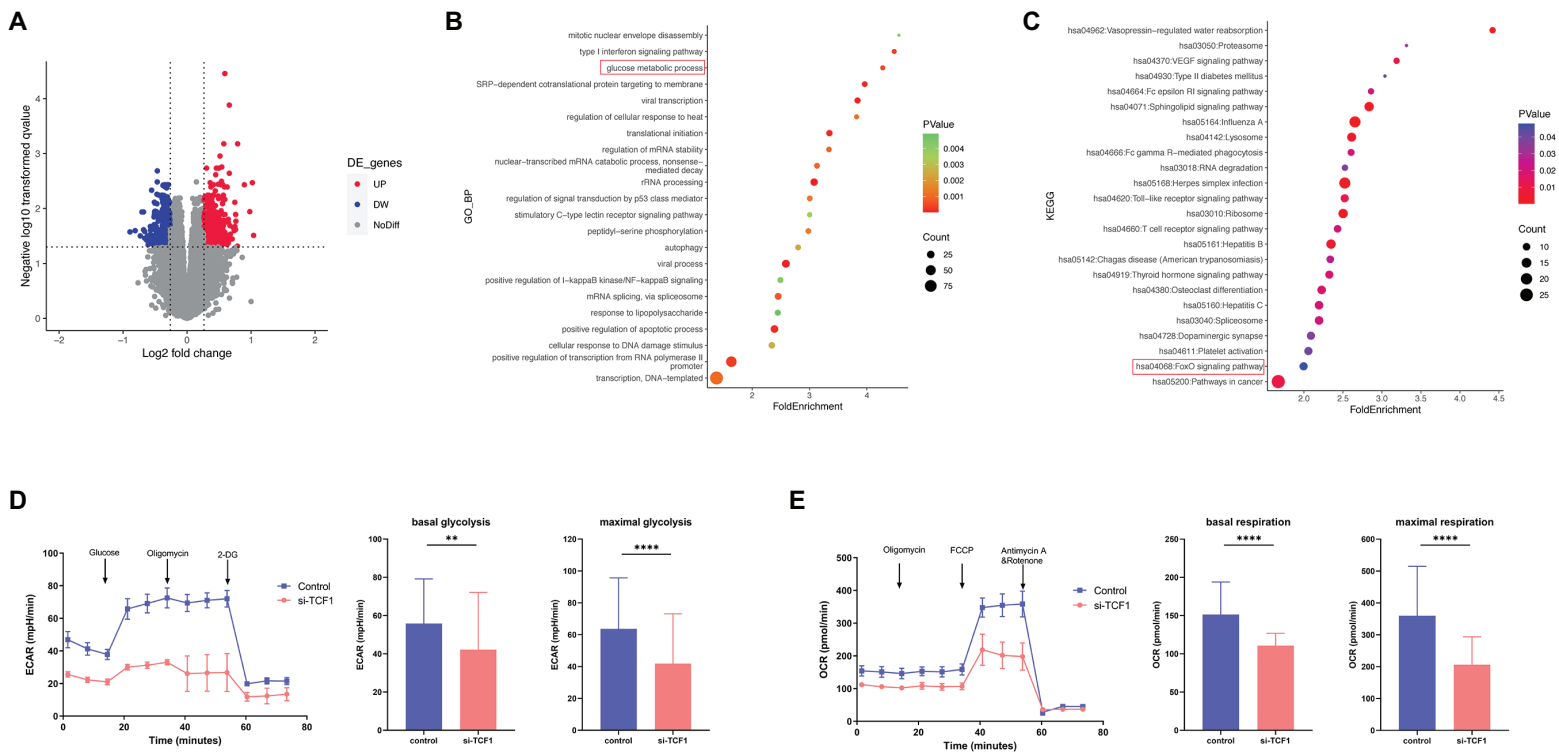
T cell factor (TCF)1 regulates the glycolytic function of T cells. **(A)** The differentially expressed genes (DEGs) in the TCF1^high^ and TCF1^low^ groups. Red points mean upregulated, and blue points mean downregulated in TCF1^high^ groups. **(B)** Gene Ontology (GO) classification of DEGs from TCF1^high^ and TCF1^low^ groups. **(C)** KEGG pathways of DEGs from TCF1^high^ and TCF1^low^ groups. **(D)** ECAR of control or TCF1 knockdown T cells were subjected using Seahorse analysis. (2-deoxyglucose = 2-DG). Basal glycolysis and maximum glycolysis capacity were calculated and compared between control and si-TCF1 group by Paired *t*-tests. **(E)** Oxygen consumption rate (OCR) at baseline and administration of oligomycin (oligo), FCCP, and Rotenone+Antimycin. Basal respiration and maximal respiration were calculated and compared between control and si-TCF1 group by Paired *t*-tests. **p < 0.01, ****p < 0.0001

To further explore the underlying mechanism of impaired cell function with TCF1, we analyzed relevant microarray data from the GEO database. According to the mean expression of TCF1 in the data, the samples were divided into two groups: TCF1^high^ and TCF1^low^. We found 693 differentially expressed genes (DEGs) with a value of *p* < 0.05 and folder change >1.2, which were used as the cutoff criteria (Figure 4A). DEGs were found to be enriched in the “glucose metabolic process” in the GO analysis (Figure 4B) and the “FoxO signaling pathway” in the KEGG analysis (Figure 4C). The FoxO signaling pathway is involved in many cellular physiological events such as glucose metabolism, so extracellular acidification rate (ECAR) assays were performed to detect changes in glycolytic function in TCF1 knockdown cells. The results showed that, compared with the control, the glycolytic function was decreased in TCF1 knockdown cells, as reflected by lower basal glycolysis (*p* = 0.0083) and maximal glycolytic capacity (*p* < 0.0001; Figure 4D). We also measured the mitochondrial respiratory function using an oxygen consumption rate (OCR) assay, and the results showed that both basal respiration (*p* < 0.0001) and maximal respiration (*p* < 0.0001) were decreased in TCF1 knockdown cells (Figure 4E). The decreases in ECAR and OCR in TCF1 knockdown cells imply that TCF1 may play a role in sustaining normal cell metabolism.

### TCF1 Knockdown Impairs Mitochondrial Function

**Figure 5:**
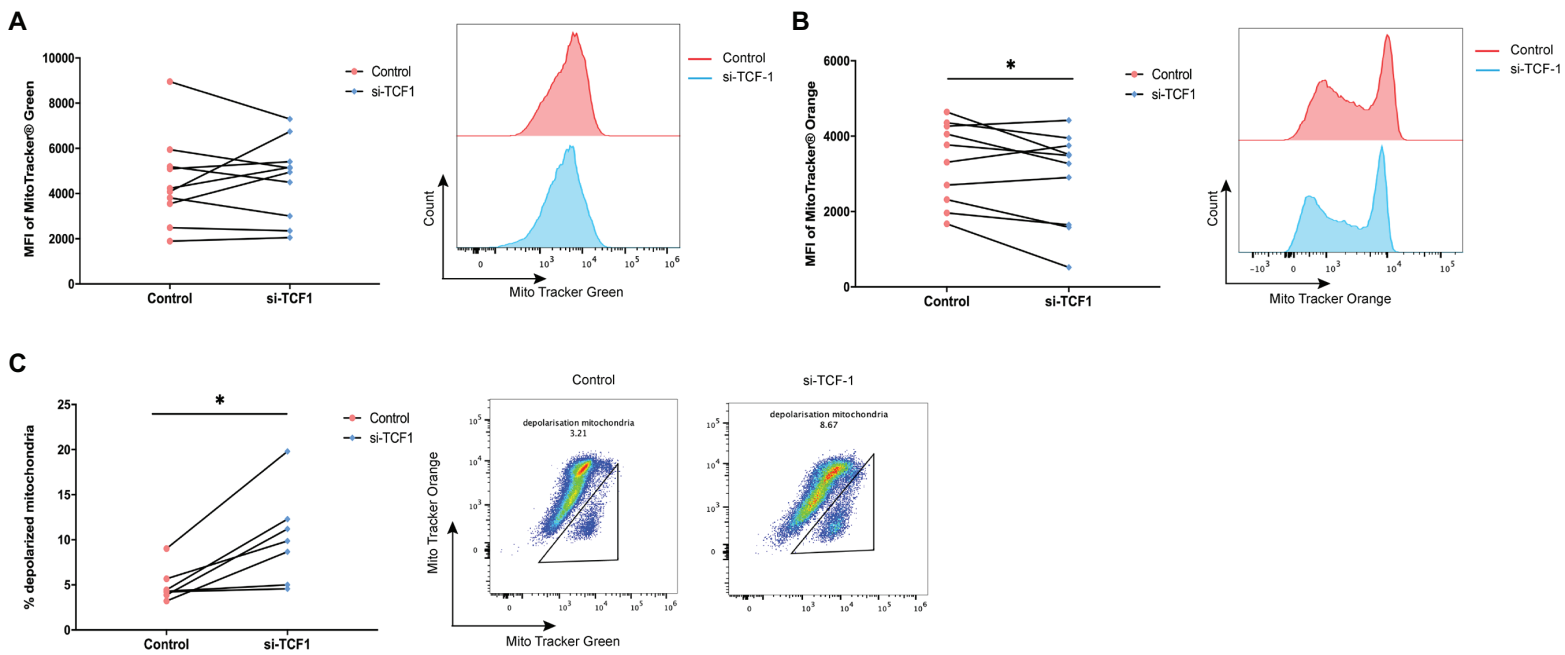
T cell factor (TCF)1 knockdown impairs mitochondrial function. After knockdown with siTCF1 for 24 h, **(A)** mitochondrial mass was analyzed in primary CD3^+^ T cells (*n* = 10) labeled with Mito Tracker Green and **(B)** mitochondrial membrane potential was analyzed in primary CD3^+^ T cells (*n* = 10) labeled with Mito Tracker Red. **(C)** The percentage of depolarized mitochondria (defined by low mitochondrial membrane potential and high mitochondrial mass [MMP^lo^/MM^hi^]) in TCF1 knockdown T cells (*n* = 7) was increased which were labeled with Mito Tracker Green and Mito Tracker Orange. Paired *t*-tests were used in **(A**-**C)**. *p < 0.05.

As mitochondria play a key role in sustaining cell function and cell metabolism, we investigated whether TCF1 downregulation impaired T-cell proliferation *via* mitochondrial dysfunction. We performed mitochondrial mass and membrane potential assays using flow cytometry in transfected T-cells. The results showed that after TCF1 knockdown, there was no change in MM (*p* = 0.7446; Figure 5A), but MMP significantly decreased compared with the control (*p* = 0.0482; Figure 5B), indicating impaired respiratory chain activity and mitochondrial function.

We observed an accumulation of depolarized mitochondria in TCF1 knockdown T-cells, characterized by increased MM but reduced MMP signals (MMP^lo^/MM^hi^; *p* = 0.0110; Figure 5C). Previously published work indicated that the MMP^lo^/MM^hi^ population usually indicates mitochondrial disruption with fewer cristae and decreased crista length, along with reductions in respiratory activity. Cells with a greater number of depolarized mitochondria had impaired effector functions (Scharping et al., 2016; Siska et al., 2017) and higher levels of PD-1 expression (Yu et al., 2020).

The above results indicate that TCF1 plays a key role in sustaining mitochondrial fitness, and the deletion of TCF1 may impair cell function *via* mitochondrial dysfunction.

## Discussion

T-cells play a pivotal role in HIV infection, affecting disease progression and therapeutic efficacy. The loss in T-cell number and dysfunction in these cells are usually accompanied by disease progression and poor clinical outcomes (Klein et al., 1995; Soghoian et al., 2012). Owing to the key role of T-cells in HIV infections, exploration of the factors affecting T-cell number and function is of great importance to reverse T-cell dysfunction for controlling HIV infections.

The change in metabolic features of T-cells during chronic infections has been explored in recent years (Bengsch et al., 2016). The consensus among researchers is that metabolism can affect T-cell development (Sun et al., 2021), proliferation (Sabharwal et al., 2018; Fultang et al., 2020), effector function (Barili et al., 2021), and exhaustion (Martins et al., 2021). Moreover, as the energy centers of cell activities, mitochondria are also critical for maintaining T-cell function (Vardhana et al., 2020; Yu et al., 2020). Mitochondria interact with the P2X1, P2X4, and P2Y11 receptors to regulate T-cell metabolism, cell migration, and antigen recognition (Ledderose and Junger, 2020). T-cell mitochondrial dysfunction can lead to premature aging (Lenaers et al., 2020), and distorted mitochondrial metabolism has a role in driving dysfunctional CD8^+^ T-cells in chronic viral infections and tumors (Leavy, 2016). TCF1, described as a stem cell-like factor in Tpex, is critical for T-cells development and effector functions and plays a crucial role in anti-tumor and antivirus activity. In our study, we analyzed the differential expression of TCF1 in HIV, ART, and NC individuals and found that lower expression of TCF1 with HIV infection may impair T-cell proliferative capacity by disrupting mitochondrial function.

In previous studies on TCF1, researchers have typically focused on CD8^+^ T-cells (Chen et al., 2021; Rutishauser et al., 2021; Zhao et al., 2021), with scant literature on the role of TCF1 in CD4^+^ T-cells. In our study, we firstly observed downregulation of TCF1 in individuals with HIV compared with NC and ART individuals, in both CD8^+^ and CD4^+^ T-cells, and confirmed a positive correlation between TCF1 downregulation and disease progression. These results indicate that TCF1 may participate in the progression of HIV infection and affect treatment outcomes. As previously reported, TCF1 is essential for memory T-cell formation, especially central memory T-cells (Jeannet et al., 2010; Zhou et al., 2010). In our study, the decline of TCF1 with HIV infection mainly occurred in memory T-cells. This result further supported the key role of TCF1 in cell differentiation. Through siRNA technology, we validated that TCF1 knockdown could impair the proliferative capacity and the production of IL2.

HIV infection is characterized by the persistence activation and inflammation (Zicari et al., 2019). Danilo et al. (2018) found that inflammatory cytokines IL-12 could downregulate TCF1 *via* IL-12R/STAT4 signaling in CD8^+^ T-cells. And increased level of the proinflammatory cytokine IL-12 was observed in HIV-infected patients (Xia et al., 2022). We speculated that a high level of proinflammation in HIV infection could contribute to the down-expression of TCF1 in T-cells. Is the downregulation of TCF1 in CD4^+^ cells a physiological process that occurs in all CD4^+^ T-cells, or is TCF1 down-regulation only in HIV-infected cells? The relationship between the HIV integration and TCF1 expression has been studied by Rutishauser et al. (2021) who did not observe a significant relationship between HIV cell-associated DNA levels in PBMCs and TCF1 expression in HIV-infected patients. Henderson et al. (2012) found that another molecule in TCF/LEF family, TCF4, represses basal HIV LTR transcription. Based on these studies, we postulated that TCF1 may not preferentially be downregulated in HIV-infected cells. It might be a decrease in both HIV-infected and non-infected cells.

Energy metabolism is required for cells to perform their functions and survive. Glycolysis and oxidative phosphorylation are two main sources of adenosine triphosphate (ATP) in cells. Lymphocyte differentiation, proliferation, and effector functions are also linked to cellular energy metabolism (Buck et al., 2017). In our results, reduced metabolism was observed in TCF1 knockdown cells, reflected by decreased OCR and ECAR; this may be caused by mitochondrial dysfunction. Owing to their ability to generate ATP for cell metabolism, mitochondria are critical for sustaining normal cellular function. Recently, Dong et al. (2021) dissected the roles of mitochondrial fusion and fission in mitochondrial function and cell proliferation, highlighting the role of mitochondria in maintaining normal cellular function and proliferative capacity. In this study, we found that the MMP significantly decreased in TCF1 knockdown T-cells and depolarized mitochondria, indicating mitochondrial dysfunction also occurred in TCF1 knockdown T-cells. Recently, Shan et al. (2022) also found the requirement for TCF1 to activate glycolysis in recall-stimulated CD8^+^ TCM cells in LCMV-infected mice. Their study showed that TCF1 activated glycolysis through the induce glycolytic enzymes by transcriptional regulation and chromatin opening in recall-stimulated CD8^+^ TCM cells, but not the canonical Wnt or NOTCH signaling pathway. We postulated that TCF1 may affect mitochondrial function and metabolism in T-cells of HIV-infected patients through signaling pathways other than Wnt or NOTCH signaling pathway, which needs further study. Our results indicate that TCF1 plays a key role in sustaining mitochondrial function, and imply that TCF1 knockdown may impair T-cell function *via* mitochondrial damage.

## Conclusion

We report that the TCF1 expression is decreased with HIV infection and TCF1 knockdown impairs T-cell function and proliferation capacity. We also validated that mitochondrial damage is caused by TCF1 knockdown, indicating that lower TCF1 expression with HIV infection could impair T-cell function *via* mitochondrial damage.

## Data Availability Statement

The original contributions presented in the study are included in the article/Supplementary Material, further inquiries can be directed to the corresponding authors.

## Ethics Statement

The studies involving human participants were reviewed and approved by the Research and Ethics Committee of the First Affiliated Hospital of China Medical University, Shenyang, China. The patients/participants provided their written informed consent to participate in this study.

## Author Contributions

HS and ZN-Z conceived and designed the experiments. HJ-C and JS carried out experiments and analyzed the data. LB-Y, JF-Z, YJ-F, and YJ-J collected the samples and contributed reagents. HS, ZN-Z, HJ-C, and JS prepared the manuscript. All authors contributed to the article and approved the submitted version.

## Funding

This work was supported by grants from the National Natural Science Foundation of China (81871708) and the Mega-Projects of National Science Research for the 13th Five-Year Plan (2017ZX10201101).

## Conflict of Interest

The authors declare that the research was conducted in the absence of any commercial or financial relationships that could be construed as a potential conflict of interest.

## Publisher’s Note

All claims expressed in this article are solely those of the authors and do not necessarily represent those of their affiliated organizations, or those of the publisher, the editors and the reviewers. Any product that may be evaluated in this article, or claim that may be made by its manufacturer, is not guaranteed or endorsed by the publisher.

## Footnotes

^1^ https://www.ncbi.nlm.nih.gov/geo/

^2^ https://www.ncbi.nlm.nih.gov/geo/geo2r/

^3^ https://david.ncifcrf.gov/tools.jsp

^4^ http://www.ehbio.com/ImageGP

## Abbreviations

ART: Antiretroviral therapy
ARTs: HIV-infected individuals receiving antiretroviral therapy
AIDS: Acquired immune deficiency Syndrome
DEGs: Differentially expressed genes
ECAR: Extracellular acidification
GEO: Gene expression omnibus
GAPDH: Glyceraldehyde 3-phosphate dehydrogenase
HIV: Human immunodeficiency virus
HIVs: Treatment-naïve HIV-infected individuals
IFN-γ: Interferon-gamma
IL2: Interleukin 2
LTNPs: Long-term non-progressors
MM: Mitochondrial mass
MMP: Mitochondrial membrane potential
NCs: HIV-Negative control individuals
OCR: Oxygen consumption rate
PBMCs: Peripheral blood mononuclear cells
qRT-PCR: Quantitative reverse-transcriptase polymerase chain reaction
ROS: Reactive oxygen species
RPs: Rapid progressors
siRNA: Small interfering RNA
TCF1: T-cell factor 1
Tex: exhausted T-cells
Tpex: Progenitor exhausted T-cells.
