# Supplementary material for: Downregulation of TCF1 in HIV Infection Impairs T-cell Proliferative Capacity by Disrupting Mitochondrial Function
Source: Front Microbiol. 2022 Jul 6;13:880873. doi: 10.3389/fmicb.2022.880873 (PMC9298517; doi:10.3389/fmicb.2022.880873)
Supplement: Supplementary file 1 [file Data_Sheet_1.docx]

<https://www.jianguoyun.com/p/DVQToRkQ_KSnChiJ8sMEIAA>

Note：Since free accounts cannot share files with anonymous users, you need to register an account before downloading files
