# Supplementary figures and images for: Downregulation of TCF1 in HIV Infection Impairs T-cell Proliferative Capacity by Disrupting Mitochondrial Function
Source: Front Microbiol. 2022 Jul 6;13:880873. doi: 10.3389/fmicb.2022.880873 (PMC9298517; doi:10.3389/fmicb.2022.880873)

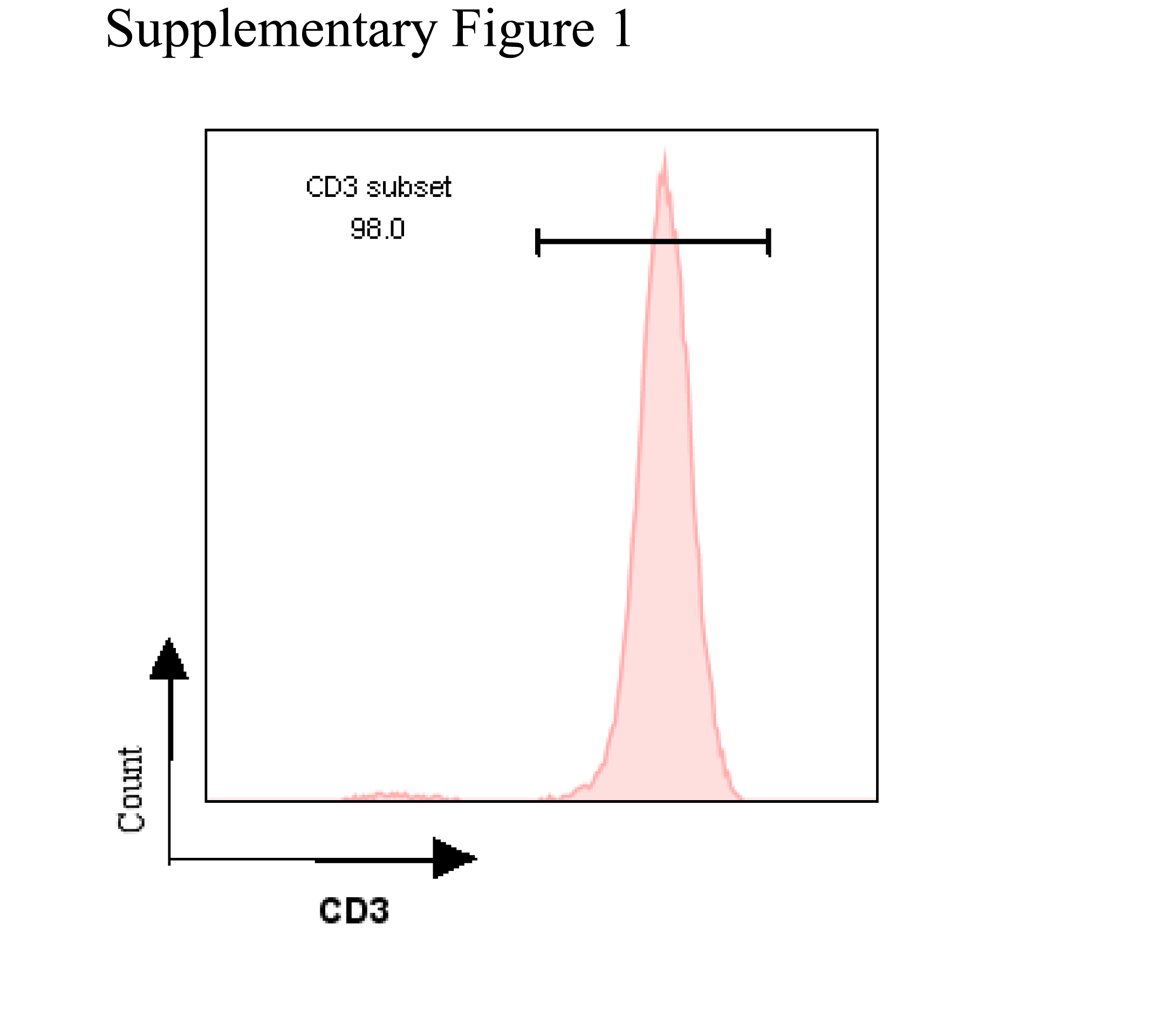

Supplement: Supplementary file 2 [file Image_1.TIF]

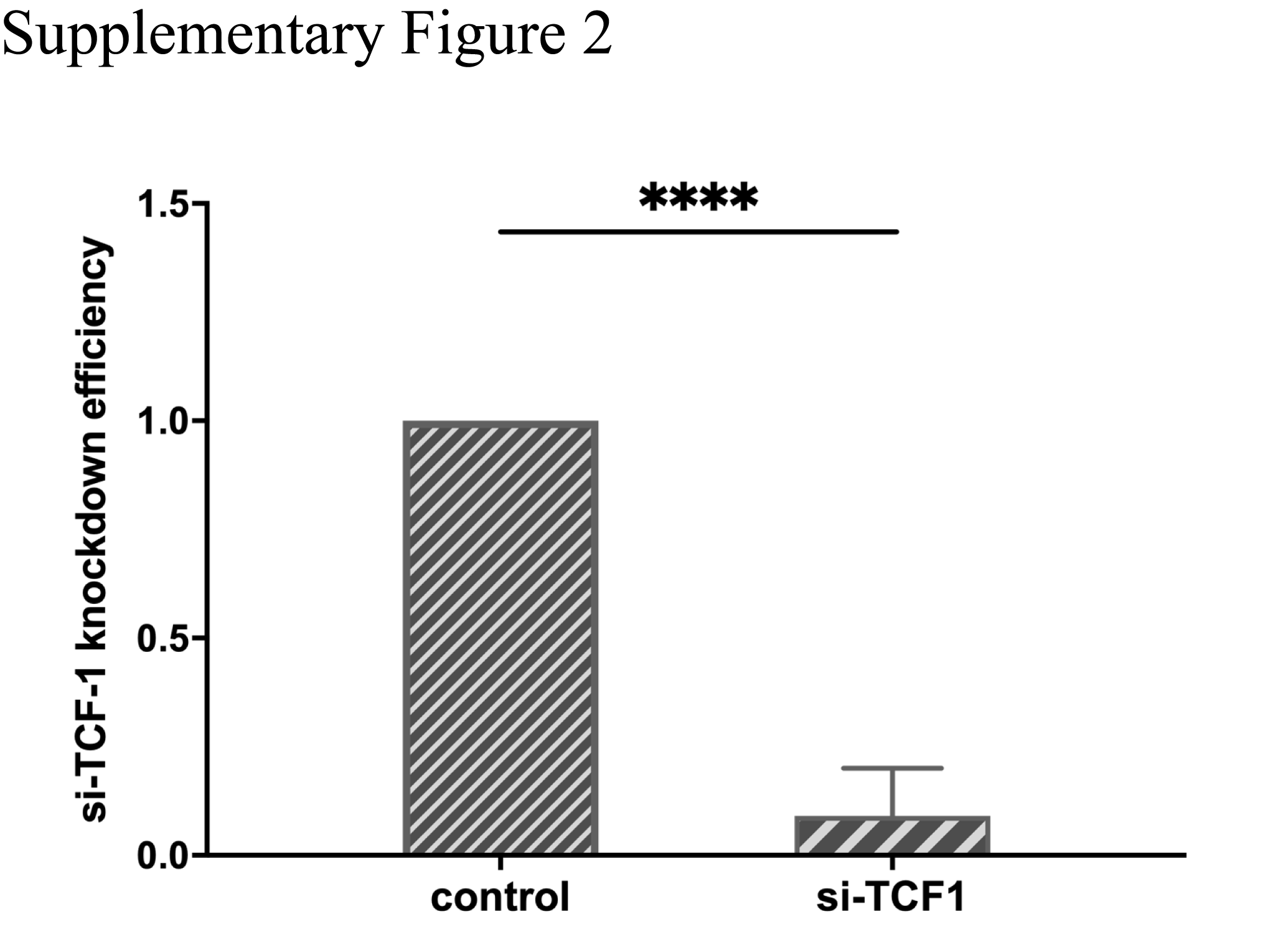

Supplement: Supplementary file 3 [file Image_2.TIF]

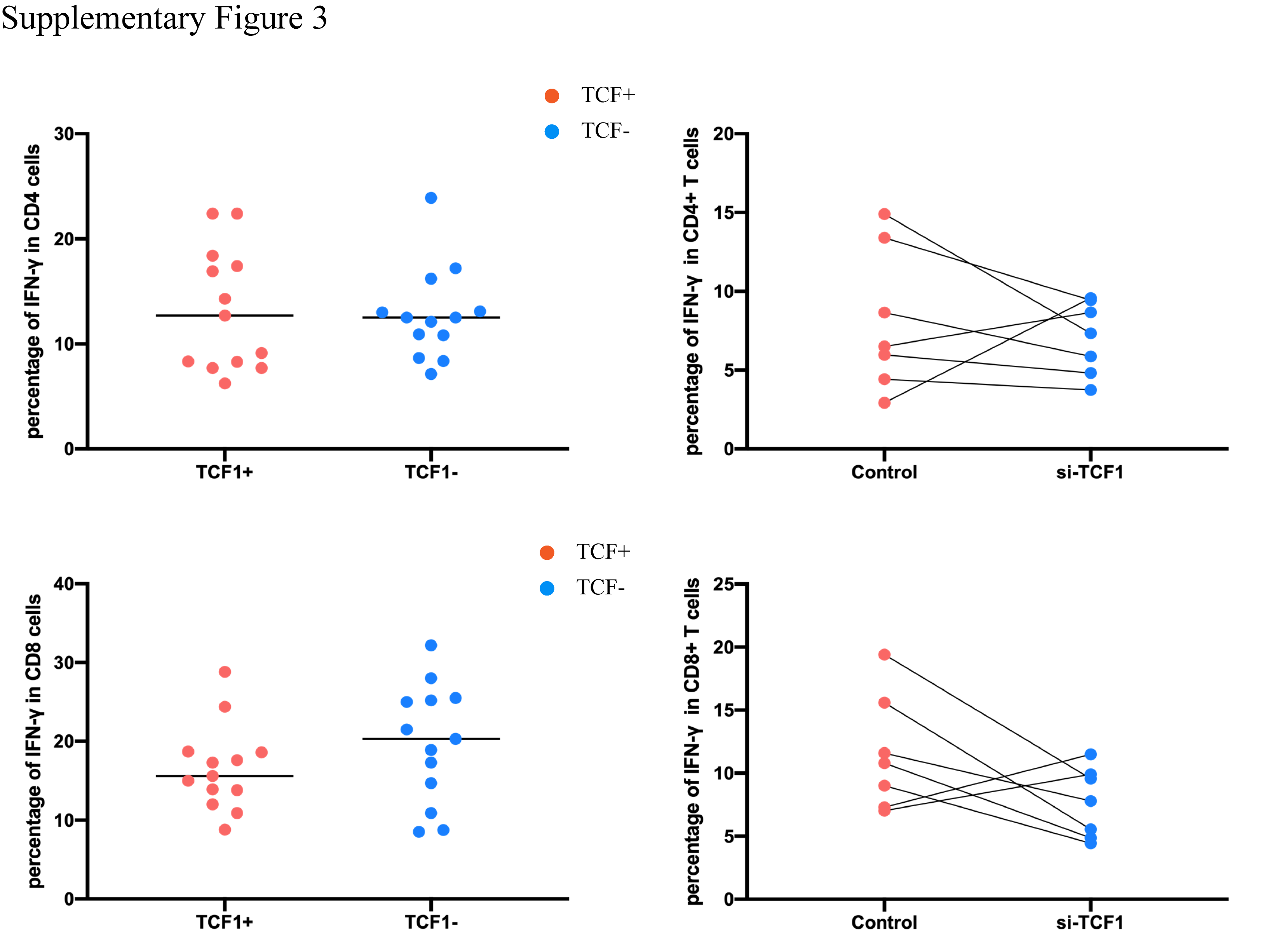

Supplement: Supplementary file 4 [file Image_3.TIF]
